# Patient and Healthcare Professional Perspectives on Knee Osteoarthritis Management in Saudi Arabia: A Qualitative Study

**DOI:** 10.7759/cureus.111877

**Published:** 2026-07-01

**Authors:** Mohammed Alnassar, Bashir Bello, Nontembiso Magida

**Affiliations:** 1 Physiotherapy Department, University of Pretoria, Pretoria, ZAF; 2 Lifestyle Disease Research Entity, North-West University, Mahikeng, ZAF; 3 Physiotherapy Department, Bayero University Kano, Kano, NGA

**Keywords:** healthcare professionals, knee osteoarthritis, patient perspectives, saudi arabia, self-management, treatment adherence

## Abstract

Background: Knee osteoarthritis (OA) is a leading cause of pain and disability worldwide, with cultural and lifestyle factors shaping its burden in different populations. In Saudi Arabia, the perspectives of both patients and healthcare professionals (HCPs) remain underexplored in understanding management approaches. This study aimed to explore patients’ and HCPs’ perspectives on management strategies for knee osteoarthritis in Saudi Arabia.

Method: A qualitative exploratory study was conducted using focus group discussions (FGDs). Participants included patients with knee OA and HCPs involved in knee OA management recruited from three hospitals within Riyadh’s First Health Cluster. FGDs were conducted separately for patients and HCPs using a semi-structured interview guide. Discussions were audio-recorded, transcribed verbatim in Arabic, and translated into English using forward-backward translation. Data were analysed inductively using reflexive thematic analysis.

Results: A total of 58 participants (30 patients and 28 HCPs) were included. Five overarching themes were identified: (i) understanding and risk factors of knee OA, (ii) clinical versus lived experience, (iii) treatment and management priorities, (iv) education and self-management, and (v) barriers and facilitators to adherence. Healthcare professionals demonstrated a biomedical understanding of knee OA, whereas patients showed variable knowledge and uncertainty. Although both groups recognised obesity and physical inactivity as key risk factors, a gap existed between knowledge and behaviour. Patients reported broader impacts on daily life and psychological well-being, which HCPs emphasised less. Divergence in treatment priorities was observed, with HCPs emphasising exercise while patients relied more on pharmacological management. Barriers to adherence included limited understanding, low motivation, and constraints in the healthcare system, while facilitators included social support and perceived improvement.

Conclusions: Knee OA management in Saudi Arabia is influenced by misalignment between clinical approaches and patient experiences, as well as cultural and system-level factors. Addressing these gaps through patient-centred, culturally tailored interventions and improved support for self-management may enhance adherence and outcomes.

## Introduction

Knee osteoarthritis (OA) is a leading cause of pain and disability worldwide and represents a major contributor to the global burden of musculoskeletal disease. Prevalence continues to increase, driven by population ageing, obesity, and physical inactivity, with knee OA accounting for the largest proportion of OA-related disability [1,2]. The condition is associated with persistent pain, reduced physical function, and impaired quality of life, and imposes substantial demands on healthcare systems [3]. International guidelines consistently recommend a core set of non-surgical interventions for knee OA, including patient education, exercise therapy, and weight management [4,5]. These approaches are effective in improving pain and function; however, their uptake in routine practice remains suboptimal. There is a well-documented evidence-practice gap, with underutilisation of first-line conservative interventions and variability in clinical management across settings [6,7]. Patients do not always follow recommended management approaches consistently, and their level of adherence is shaped by personal circumstances as well as broader contextual influences.

Given the chronic and progressive nature of knee OA, long-term management relies heavily on self-management. Patients are expected to engage in sustained behaviours, including regular physical activity, symptom monitoring, and lifestyle modification. Although self-management interventions are associated with modest improvements in clinical outcomes, their effectiveness depends on patient engagement, health literacy, and the extent to which care is tailored to individual circumstances [8]. Patients frequently report uncertainty regarding disease progression, inconsistent information, and difficulty maintaining behavioural changes over time [9]. Healthcare professionals (HCPs) play a central role in supporting self-management and delivering evidence-based care. Effective management requires clear communication, shared decision-making, and coordination across disciplines. However, variation in clinician knowledge, attitudes, and practices, as well as constraints on time and resources, may limit the implementation of guideline-recommended care [10]. Misalignment between patient expectations and provider recommendations may further contribute to suboptimal outcomes.

In Saudi Arabia, the burden of knee OA is increasing in parallel with demographic and lifestyle changes, including rising rates of obesity and reduced physical activity [11]. Sociocultural factors may influence both health behaviours and engagement with care, including attitudes toward exercise, gender-related norms, and the role of family in health decision-making. Similarly, the healthcare system is undergoing reform, with increasing emphasis on prevention and patient-centred care. Despite this, there is limited evidence describing how knee OA is managed in routine practice within this context. Qualitative studies conducted in other settings have identified a range of strategies used by patients to manage knee OA, including activity modification, pharmacological treatments, and engagement in rehabilitation, as well as barriers such as pain-related fear and limited access to services. From the provider's perspective, challenges include promoting adherence to lifestyle interventions and addressing patient expectations for medical or surgical management [12-14]. However, few studies have examined both patient and HCP perspectives within the same study, and evidence from Middle Eastern populations remains limited. Hence, understanding how management strategies are adopted and sustained within specific cultural and healthcare contexts is important for informing the design and implementation of effective interventions. In particular, examining areas of alignment and divergence between patient and provider perspectives may help to improve communication, enhance adherence, and optimise care delivery. The aim of this study is, therefore, to explore patients’ and HCPs’ perspectives on management strategies for knee osteoarthritis in Saudi Arabia.

## Materials and methods

A qualitative exploratory study design was employed, using focus group discussions (FGDs) to explore the perspectives of patients and HCPs on knee OA management strategies in Saudi Arabia. The study was conducted at Al-Iman General Hospital, King Salman Hospital, and King Saud Medical City, all located within Riyadh’s First Health Cluster, Saudi Arabia. These facilities provide multidisciplinary care for individuals with musculoskeletal conditions, including knee OA. The study is reported in accordance with the Consolidated Criteria for Reporting Qualitative Research (COREQ) [15].

The study was approved by the Research Ethics Committee of the Faculty of Health Science, University of Pretoria, Pretoria, South Africa (ethics reference number: 581/2024 - Line 5). Permission was granted by Al-Iman General Hospital, King Salman Hospital, and King Saud Medical City. All participants provided written informed consent prior to participation. Confidentiality and anonymity were maintained by de-identifying all transcripts and using coded identifiers. Data were securely stored and accessible only to the research team.

Study population

Participants were purposively recruited to include individuals with relevant experience in knee OA management. Two groups were included: (i) HCPs involved in the management of knee OA (e.g., physiotherapists and physicians), and (ii) patients with a clinical diagnosis of knee OA. HCPs were recruited through the participating institutions and professional networks, while patients were recruited from outpatient and rehabilitation clinics within the study sites. Eligible patients were adults with a confirmed diagnosis of knee OA and the ability to participate in group discussions. Participants were approached directly or through institutional contacts and provided with study information prior to enrolment. Written informed consent was obtained from all participants.

Data collection

Data were collected through separate FGDs with HCPs and patients to facilitate open, homogeneous group discussions. The FGDs were conducted by a moderator with formal training in qualitative research and experience in musculoskeletal rehabilitation. The moderator had no prior clinical relationship with the participants. A note-taker was present during all sessions to document non-verbal cues and contextual observations. To minimise bias, reflexive notes were maintained throughout data collection and analysis, and assumptions were discussed within the research team.

A total of five FGDs were conducted with HCPs and five with patients, each group comprising at least five participants. A FGD guide was developed based on existing literature and the study objectives (see Appendices). The guide explored experiences of knee OA management, strategies used in daily practice or self-management, perceived barriers and facilitators, and interactions with the healthcare system. The guide was pilot-tested and refined prior to use. FGDs were conducted in a private setting convenient for participants and lasted approximately 60-90 minutes. All discussions were audio-recorded with participants’ permission. Field notes were taken to capture contextual information and group interactions. Data collection continued until data saturation was reached, defined as the point at which no new codes or themes emerged from subsequent discussions.

To ensure the accuracy and integrity of the translation process, a rigorous forward-backward translation approach was employed. All FGDs were initially transcribed verbatim in Arabic to preserve the original meaning and contextual nuances. The transcripts were then translated into English by a bilingual translator with expertise in both languages and familiarity with the study context. Subsequently, an independent bilingual translator, blinded to the original transcripts, performed back-translation of the English version into Arabic. The original and back-translated Arabic versions were compared systematically to identify discrepancies in meaning, terminology, and interpretation. Any inconsistencies were reviewed and resolved through discussion among the translators and the research team until consensus was achieved. Particular attention was paid to culturally specific expressions and idiomatic language to ensure conceptual equivalence rather than literal translation. This iterative process enhanced the linguistic accuracy, credibility, and trustworthiness of the data prior to analysis.

Data analysis

Data were analysed using reflexive thematic analysis as described by Braun and Clark [16]. An inductive approach was adopted to generate themes from the data rather than being driven by pre-existing frameworks.

The analysis followed the six-phase process outlined by Braun and Clark [16]. First, transcripts were read and re-read to achieve familiarisation with the data, alongside listening to audio recordings where necessary to ensure accuracy and depth of understanding. Initial ideas and observations were documented during this phase. Second, initial codes were generated systematically across the dataset. Meaningful segments of text relevant to the research question were identified and labelled. Coding was conducted using Atlas.ti (https://atlasti.com/) to facilitate data organisation and retrieval. Third, codes were examined and collated into potential themes by identifying patterns and relationships across the dataset. Related codes were grouped into broader categories. Fourth, candidate themes were reviewed and refined through an iterative process. This involved checking the coherence of coded extracts within each theme and ensuring that themes accurately reflected the dataset as a whole. Themes were revised, merged, or discarded where necessary. Fifth, themes were defined and named. Clear definitions were developed for each theme and subtheme, capturing the essence of the data they represented and their relevance to the study objectives. Finally, the analysis was synthesised and reported. Representative verbatim quotations were selected to illustrate key themes and to retain the participants’ voices.

To enhance analytical rigour, coding and theme development were conducted independently by two researchers (MA and NM), followed by regular discussions to compare interpretations and resolve discrepancies. An iterative and reflexive approach was maintained throughout the analysis to ensure that findings were grounded in the data. In line with reflexive thematic analysis, we considered our positionality throughout the study, recognising that our disciplinary training and cultural context influenced theme development. Trustworthiness was ensured through several strategies. Credibility was enhanced through prolonged engagement with the data, verbatim transcription, and investigator triangulation. Dependability was supported by maintaining an audit trail of coding decisions and theme development. Confirmability was addressed through reflexive journaling and independent coding. Transferability was facilitated by providing detailed descriptions of the study context, participants, and data collection procedures.

## Results

A total of 58 participants were included in the study, comprising 30 patients and 28 HCPs. Patients were predominantly female (70%) and ranged in age from 25 to 84 years, with most aged 45 years or older. The duration of knee OA ranged from two to 20 years. Participants represented diverse occupational backgrounds. HCPs included physiotherapists, rehabilitation consultants, physiatrists, and consultant orthopaedic surgeons, with professional experience ranging from three to 25 years. Most HCPs were based in outpatient settings. Participant characteristics are summarised in Table 1.

**Table 1:** Sociodemographic characteristics of the participants (N=58) HCP: healthcare professional

Characteristic	Patients (n = 30)	HCPs (n = 28)
Gender, n (%)	Females: 21 (70%)	10 (36%)
	Males: 9 (30%)	18 (64%)
Age (years), range	25 - 84	28 - 64
Occupation / Profession	Retired	Physiotherapists
	Unemployed	Physiatrists
	Teachers	Rehabilitation consultants
	Office workers	Orthopaedic surgeons
	Manual workers	
Disease duration (years), range	2 - 20	—
Professional experience (years), range	—	3 - 25
Knee OA-specific experience (years), range	—	2 - 21

Thematic analysis

Thematic analysis generated five overarching themes reflecting integrated perspectives of patients and HCPs: (i) understanding and risk factors of knee OA, (ii) clinical versus lived experience, (iii) treatment and management priorities, (iv) education and self-management, and (v) barriers and facilitators to adherence. An overview of these themes, associated subthemes, and representative quotations is presented in Table 2. 

**Table 2:** Themes, subthemes, and representative quotations on the management of knee osteoarthritis in Saudi Arabia HCP: healthcare professional

Theme	Subtheme	Interpretive description	Representative quotations
Understanding and risk factors of knee OA	Knowledge of condition	Variation in understanding between HCPs and patients	“Most of our patients are overweight or obese” (HCP); “I don’t know the cause” (Patient)
	Modifiable risk factors	Recognition of obesity and inactivity	“Higher BMI correlates with more symptoms” (HCP); “My knee worsened with weight gain” (Patient)
	Cultural practices	Lifestyle influences such as floor sitting	“Floor sitting increases knee stress” (HCP); “Sitting on the floor is painful” (Patient)
Clinical versus lived experience	Physical symptoms	Pain and functional limitation	“Pain during stairs and walking” (HCP); “I cannot walk long distances” (Patient)
	Impact on daily life	Effect on daily and religious activities	“I pray on a chair” (Patient)
	Psychological impact	Fear and perceived disability	“I am afraid of falling” (Patient); “It feels like becoming partially disabled” (Patient)
Treatment and management priorities	Exercise-based management	Exercise prioritised but inconsistently followed	“Exercise is essential” (HCP); “I stop exercises” (Patient)
	Pharmacological reliance	Preference for medication vs clinical caution	“I use painkillers” (Patient); “Patients overuse painkillers” (HCP)
	Individualised care	Tailored care not always perceived	“Each patient needs a tailored plan” (HCP)
Education and self-management	Structured education	HCP-led education strategies	“We educate patients on weight reduction” (HCP)
	Informal information sources	Use of online/social media sources	“I search online” (Patient)
	Self-management behaviours	Variable adherence to strategies	“I lost weight and improved” (Patient)
Barriers and facilitators to adherence	Knowledge barriers	Limited understanding and confusion	“Patients lack understanding” (HCP); “I am confused” (Patient)
	Behavioural barriers	Poor adherence and motivation	“Patients stop exercises” (HCP); “I don’t continue” (Patient)
	System-level barriers	Time and access constraints	“We have limited time” (HCP); “Appointments are hard to get” (Patient)
	Facilitators	Social support and perceived improvement	“Family supports me” (Patient); “When I improve, I continue” (Patient)

Understanding the Risk Factors of Knee OA

HCPs demonstrated a consistent biomedical understanding of knee OA, emphasising ageing, obesity, and physical inactivity as key risk factors. One HCP noted, “Most of the patients we see are over 50 years” (HCP, G5P2), while another stated, “Most of our patients are overweight or obese” (HCP, G2P1). In contrast, patients showed variable understanding, with some attributing symptoms to ageing, “It is due to ageing” (Patient, G1P7), while others expressed uncertainty, “I don’t know the cause” (Patient, G2P3). There was strong agreement regarding the role of obesity, with a patient reporting, “My knee worsened with weight gain” (Patient, G2P4), consistent with HCP observations that higher body mass is associated with increased symptom severity. Cultural practices were also identified, with one HCP noting, “Floor sitting increases knee stress” (HCP, G1P1), while a patient added, “Sitting on the floor is painful” (Patient, G2P1). These patterns are reflected across the subthemes presented in Table 2.

Clinical Versus Lived Experience

HCPs described knee OA primarily in terms of pain and functional limitations, such as “They complain of pain during stairs and walking” (HCP, G2P1). In contrast, patients described a broader lived experience, including substantial functional restriction: “I cannot walk long distances” (Patient, G4P5). Daily activities and religious practices were notably affected. One participant stated, “I pray on a chair” (Patient, G1P2), reflecting adaptations required for daily functioning. Psychological impacts were also prominent among patients, with expressions such as “I am afraid of falling” (Patient, G2P3) and “It feels like becoming partially disabled” (Patient, G4P4). These dimensions were less emphasised by HCPs, highlighting a gap in addressing psychosocial aspects of care. Table 2 further highlights these differences.

Treatment and Management Priorities

HCPs consistently identified exercise as the cornerstone of management. As one HCP explained, “Exercise is essential for management” (HCP, G1P2). However, patient adherence was inconsistent. While some participants acknowledged benefits, others reported discontinuation: “I stop exercising” (Patient, G4P2). Patients frequently prioritised pharmacological management, with statements such as “I use painkillers” (Patient, G4P6), whereas HCPs expressed concern regarding over-reliance on medication, noting that patients often depend on analgesics for symptom control. Although HCPs emphasised individualised care, “Each patient needs a tailored plan” (HCP, G4P3), this was not consistently perceived by patients, indicating a communication gap. These contrasts are illustrated in Table 2.

Education and Self-Management

HCPs described providing structured education focused on lifestyle modification and adherence, for example, “We educate patients on weight reduction” (HCP, G4P1). However, patients reported variability in the information they received and often relied on informal sources, such as “I searched online” (Patient, G2P3). Confusion due to conflicting advice was also evident, with one participant stating, “Too many opinions… I don’t know who to follow” (Patient, G4P4). While some patients demonstrated effective self-management (“I lost weight and improved” (Patient, G4P4)), others reported inconsistent adherence. We recognise that our professional training may have led us to emphasise clinical management aspects more strongly than patients’ everyday experiences. A detailed breakdown of these findings is provided in Table 2.

Barriers and Facilitators to Adherence

Barriers to adherence were identified at multiple levels. HCPs highlighted knowledge gaps: “Patients lack understanding of management” (HCP, G4P3), while patients expressed confusion: “I am confused” (Patient, G4P4). Behavioural barriers were also prominent, with patients acknowledging, “I don’t continue” (Patient, G4P2). System-level challenges were reported, including limited access and time constraints, with one patient noting, “Appointments are hard to get” (Patient, G4P3), and HCPs indicating limited time for education. Facilitators included social support and perceived improvement, as reflected in the statement, “Family supports me” (Patient, G2P4). Overall, these patterns are consistent with the thematic structure outlined in Table 2.

## Discussion

This study explored the strategies used by patients and HCPs to manage knee OA in Saudi Arabia. The findings highlight important gaps between clinical knowledge and patient experience, inconsistencies in treatment priorities, and multilevel barriers influencing adherence. Collectively, the results underscore the need for more patient-centred, contextually tailored approaches to knee OA management. A key finding was the disparity in understanding of knee OA between patients and HCPs. While HCPs demonstrated a consistent biomedical perspective, patients showed variable and, at times, limited understanding of disease aetiology. This aligns with previous qualitative research indicating that patients often attribute OA to ageing or remain uncertain about its causes, which may reduce engagement in preventive and self-management behaviours [9]. Notably, both groups recognised obesity and physical inactivity as major risk factors, reflecting increasing awareness of modifiable contributors to disease progression [2]. However, the persistence of sedentary behaviours despite this awareness highlights a well-documented knowledge-behaviour gap in OA management [10]. Cultural practices, such as floor sitting, emerged as context-specific contributors to symptom exacerbation, emphasising the importance of culturally sensitive recommendations.

The distinction between clinical and lived experience was particularly evident. HCPs focused primarily on pain and functional limitations, whereas patients described a broader impact encompassing daily activities, religious practices, and psychological well-being. The limited emphasis on psychological factors by HCPs is consistent with previous reports that psychosocial dimensions of OA are often under-recognised in routine care [17]. Cross and colleagues affirmed that the fear of falling and perceived disability, as reported by patients, are known to contribute to activity avoidance and functional decline [3]. These findings suggest that current models of care may insufficiently address the biopsychosocial nature of knee OA.

Divergence in treatment priorities was also evident. HCPs consistently identified exercise as the cornerstone of management, in line with international guidelines [4,5,18]. However, patient adherence to exercise was inconsistent, with many participants discontinuing home-based programmes. This reflects a persistent challenge in OA care, where exercise adherence remains suboptimal despite strong evidence of benefit [8,19]. In contrast, patients frequently prioritised pharmacological management, often relying on analgesics to maintain function. This mismatch between recommended care and patient preference has been widely reported and may reflect immediate symptom relief, limited understanding of long-term management strategies, and insufficient reinforcement of non-pharmacological approaches [7].

Education and self-management practices were variable across participants. Although HCPs reported providing structured education, patients frequently described inconsistent or insufficient information, often supplemented by informal sources such as online platforms. This reliance on non-clinical information sources may contribute to confusion and conflicting beliefs about management, as observed in this study. Previous evidence suggests that effective self-management requires not only knowledge provision but also ongoing support, behavioural reinforcement, and clear communication [11]. The variability observed here indicates gaps in the delivery and uptake of patient education within routine care.

Barriers to adherence were identified at multiple levels, including knowledge, behaviour, and healthcare system factors. In their systematic review, Smith et al. found that limited understanding, low motivation, and difficulty sustaining behavioural change were prominent patient-related barriers, consistent with the existing literature [9]. They also mentioned that system-level constraints, including limited consultation time and restricted access to services, further hindered effective management. Importantly, facilitators such as social support and perceived improvement were identified as key drivers of adherence. The role of family support is particularly relevant in the Saudi context, where family structures play a central role in health behaviours and decision-making. Research in Saudi Arabia highlights the critical role of family members as informal caregivers, who are often responsible for escorting patients to appointments, managing medications, and providing emotional support. Family structures are deeply embedded in healthcare decision-making, reflecting cultural and social norms in the region [20]. Taken together, these findings highlight the need to move beyond a purely biomedical model toward a more integrated, patient-centred approach to knee OA management. Interventions should address not only physical symptoms but also psychological, social, and cultural factors influencing behaviour. Strategies to improve adherence should incorporate behavioural change techniques, structured follow-up, and culturally relevant education. Enhancing communication between patients and HCPs and aligning treatment priorities may also improve engagement and outcomes.

A key strength of this study is the inclusion of both patient and HCP perspectives, allowing for the identification of areas of convergence and divergence in knee OA management. The use of focus group discussions facilitated rich data and interaction among participants. However, the study is limited to a single health cluster in Riyadh, which may affect transferability. Additionally, as with all qualitative research, findings are context-specific and may not be generalisable to other settings.

## Conclusions

The findings underscore the need for contextually tailored, multidisciplinary approaches to knee OA management in Saudi Arabia that actively prioritise patient engagement and self‑management. Addressing the mismatch between clinical priorities and patient experiences requires interventions that are not only evidence‑based but also culturally adapted to local practices and values. Future research should therefore focus on the development and rigorous evaluation of culturally sensitive strategies that mitigate identified barriers while leveraging facilitators such as social support networks. Moreover, the study highlights the importance of strengthening the implementation of guideline‑recommended care within routine clinical practice. For instance, patients in our study emphasised the importance of family involvement and culturally appropriate education in supporting adherence to conservative management strategies.

Incorporating these perspectives into guideline implementation could help bridge the evidence‑practice gap by ensuring that recommended interventions are delivered in ways that resonate with patients’ experiences. Bridging the gap between policy and practice will demand strategies that integrate patient perspectives, enhance adherence, and foster collaboration across disciplines. For example, patient feedback on the need for culturally appropriate education and family support could be incorporated into guideline development and multidisciplinary team discussions, ensuring that recommendations are implemented in ways that resonate with patients in real-world practice. By embedding patient‑centred and culturally responsive approaches into everyday care, healthcare systems may achieve improved outcomes, greater sustainability of self‑management behaviours, and enhanced quality of life for individuals living with knee OA.

## Appendices

Focus group discussion guide: healthcare professionals

1. Can you describe your experience in managing patients with knee osteoarthritis in your clinical setting? (Probe if needed - common patient profile, severity and progression)

2. From your experience, what factors most influence the development and progression of knee osteoarthritis among your patients?

3. How do you currently manage knee osteoarthritis, and what strategies do you prioritise in practice?

4. How do you educate and support patients to manage their knee osteoarthritis? (Probe: mode of delivery, e.g., individual or group; follow-up?)

5. What factors help or hinder effective education and long-term self-management for patients with knee osteoarthritis? (Probe - ask them to expand patient-related, Healthcare system-related, or Cultural or contextual factors)

6. What are your views on developing an educational programme for the secondary prevention of knee osteoarthritis in Saudi Arabia? (Probe - what is the key content? Structure and delivery, and/ or Feasibility and sustainability)

7.  What recommendations would you make to improve education and secondary prevention of knee osteoarthritis?

Focus group discussion guide: patients with knee osteoarthritis

1. Can you describe your experience of living with knee osteoarthritis? (Probe - Symptoms, Daily challenges and/or Emotional impact)

2. What do you believe caused your knee osteoarthritis, and how do you understand your condition? (Probe: Weight, activity levels, Age, injuries, Health conditions)

3. How does knee osteoarthritis affect your daily activities and quality of life? (Probe: Work, Mobility, Social participation)

4. How do you currently manage your knee osteoarthritis? (Probe: Exercise, Weight management, Medication, Home strategies)

5. What education or advice have you received about managing knee osteoarthritis, and how helpful was it? (Probe: Type of information, Mode of delivery, Follow-up support)

6. What makes it difficult or easier for you to manage your knee osteoarthritis? (Probe: Motivation, Support systems, Cultural or environmental factors)

7.  What would an ideal education programme for knee osteoarthritis look like for you? (Topics, Format, Follow-up and support, Cultural suitability)

8. Is there anything else you would like to share about managing knee osteoarthritis?
